# Quality assurance of single isocentre multiple target stereotactic radiosurgery: Findings from long‐term off‐axis Winston‐Lutz testing and machine performance checks

**DOI:** 10.1002/acm2.70275

**Published:** 2025-09-30

**Authors:** Joel Poder, Robert Chambers, Harry Porter, Ryan Brown, Dylan Koprivec, Johnson Yuen, Rebecca Quezeda, Lisa Nourbehesht, Andrew Howie

**Affiliations:** ^1^ St George Hospital Cancer Care Centre Kogarah NSW Australia; ^2^ Centre for Medical Radiation Physics University of Wollongong Wollongong NSW Australia; ^3^ Institute of Medical Physics University of Sydney Camperdown NSW Australia; ^4^ St. George and Sutherland Clinical School University of New South Wales Kogarah NSW Australia

**Keywords:** off‐axis, quality assurance, stereotactic radiosurgery, Winston‐Lutz

## Abstract

**Background:**

Single‐isocenter multiple‐target stereotactic radiosurgery (SIMT SRS) has emerged as an efficient treatment for multiple brain metastases. However, this technique demands exceptional geometric accuracy, particularly off‐axis, to ensure optimal dose delivery while sparing healthy tissue. Traditional quality assurance (QA) methods require adaptation for SIMT SRS, highlighting the need for robust testing protocols.

**Purpose:**

This study aimed to evaluate the long‐term off‐axis targeting accuracy of three Varian TrueBeam linear accelerators using a dedicated off‐axis Winston–Lutz test (OAWLT) and to investigate correlations with routine varian machine performance check (MPC) results.

**Materials & Methods:**

Weekly OAWLT measurements were performed over a 6‐month period on three TrueBeam linacs using the Sun Nuclear StereoPHAN phantom and MultiMet‐WL cube. The test delivered 6 MV flattening filter‐free beams to multiple off‐axis targets via eight beam orientations, simulating clinical SIMT SRS workflows. Concurrently, daily MPC assessments evaluated geometric parameters including isocenter size, kV isocenter offset, beam center, and couch radiation‐induced offset.

**Results:**

Median OAWLT errors were 0.38, 0.44, and 0.59 mm for the three linacs included in this study, with maximum errors of 1.12, 1.08, and 1.54 mm. Notably, off‐axis errors increased with target off‐axis distance, with the worst performance observed at 7 cm off‐axis.

**Conclusion:**

Routine OAWLT is an effective and sensitive QA tool for monitoring off‐axis targeting accuracy in SIMT SRS. Despite the convenience of daily MPC, it cannot substitute for a dedicated OAWLT. The study supports implementing weekly OAWLT in clinical practice to ensure high‐quality, consistent treatment delivery for SIMT SRS treatments.

## 1 INTRODUCTION

Stereotactic radiosurgery (SRS) is a highly precise radiation therapy technique used to deliver high doses of radiation to intracranial targets while minimizing exposure to surrounding healthy tissue.^1^ The success of SRS relies on achieving steep dose gradients and sub‐millimeter targeting accuracy. Traditionally, SRS has been employed to treat single targets using a single isocenter, with a well‐established quality assurance (QA) framework to ensure geometric accuracy.^1^

However, the growing incidence of brain metastases has led to a paradigm shift toward single‐isocenter, multiple‐target (SIMT) SRS.^2^ This approach offers significant advantages in clinical efficiency by reducing treatment time and patient repositioning, but it also presents new challenges.^3^ Unlike single‐target SRS, SIMT SRS requires precise dose delivery not only at the isocenter but also across off‐axis regions, where geometric accuracy becomes more susceptible to uncertainties due to mechanical and dosimetric factors.^4^, ^5^ As a result, robust QA protocols are essential to ensure safe and effective SIMT SRS delivery.^6^, ^7^

Quality assurance for SIMT SRS is an evolving field, with ongoing efforts to develop and refine tests that can reliably assess geometric accuracy. The off‐axis Winston‐Lutz test (OAWLT) is a test designed specifically to assess central axis and off‐axis targeting accuracy. It provides direct measurements of positional deviations at multiple off‐axis locations, offering a more comprehensive evaluation compared to traditional Winston‐Lutz tests.^6^, ^7^ Varian's Machine Performance Check (MPC), an automated daily QA tool that evaluates various mechanical and dosimetric parameters of TrueBeam linear accelerators (linacs).^8^, ^9^ Although not specifically designed for SIMT SRS, MPC data may offer insights into long‐term trends in machine performance that pertain to SIMT SRS treatments.^10^, ^11^

Despite established QA protocols and growing interest in improving geometric accuracy for SIMT SRS, significant uncertainties remain.^12^ The long‐term consistency of off‐axis targeting accuracy across multiple linacs has not been thoroughly studied. Most available data focus on short‐term results or isolated cases,^13^ leaving a gap in understanding how machine performance evolves over extended periods as pertaining to off‐axis targeting accuracy. Moreover, the potential correlation between routine MPC data and OAWLT results is not well‐characterized, particularly for SIMT SRS applications.^14^, ^15^ Establishing this relationship could enhance the predictive value of MPC and streamline QA processes by identifying early signs of performance drift.

This study aims to address these gaps by characterizing the long‐term accuracy of three Varian TrueBeam linear accelerators in delivering SIMT SRS over a 6 month period. Additionally, the study will investigate the relationship between OAWLT results and long‐term MPC data, evaluating the role of MPC in comprehensive QA for SIMT SRS. By providing long‐term data across multiple machines, this study seeks to offer practical insights into improving QA strategies and ensuring consistent, high‐quality treatment delivery for patients undergoing SIMT SRS.

## 2 METHODS

### 2.1 StereoPhan and multiMet Winston‐Lutz cube

**FIGURE 1:**
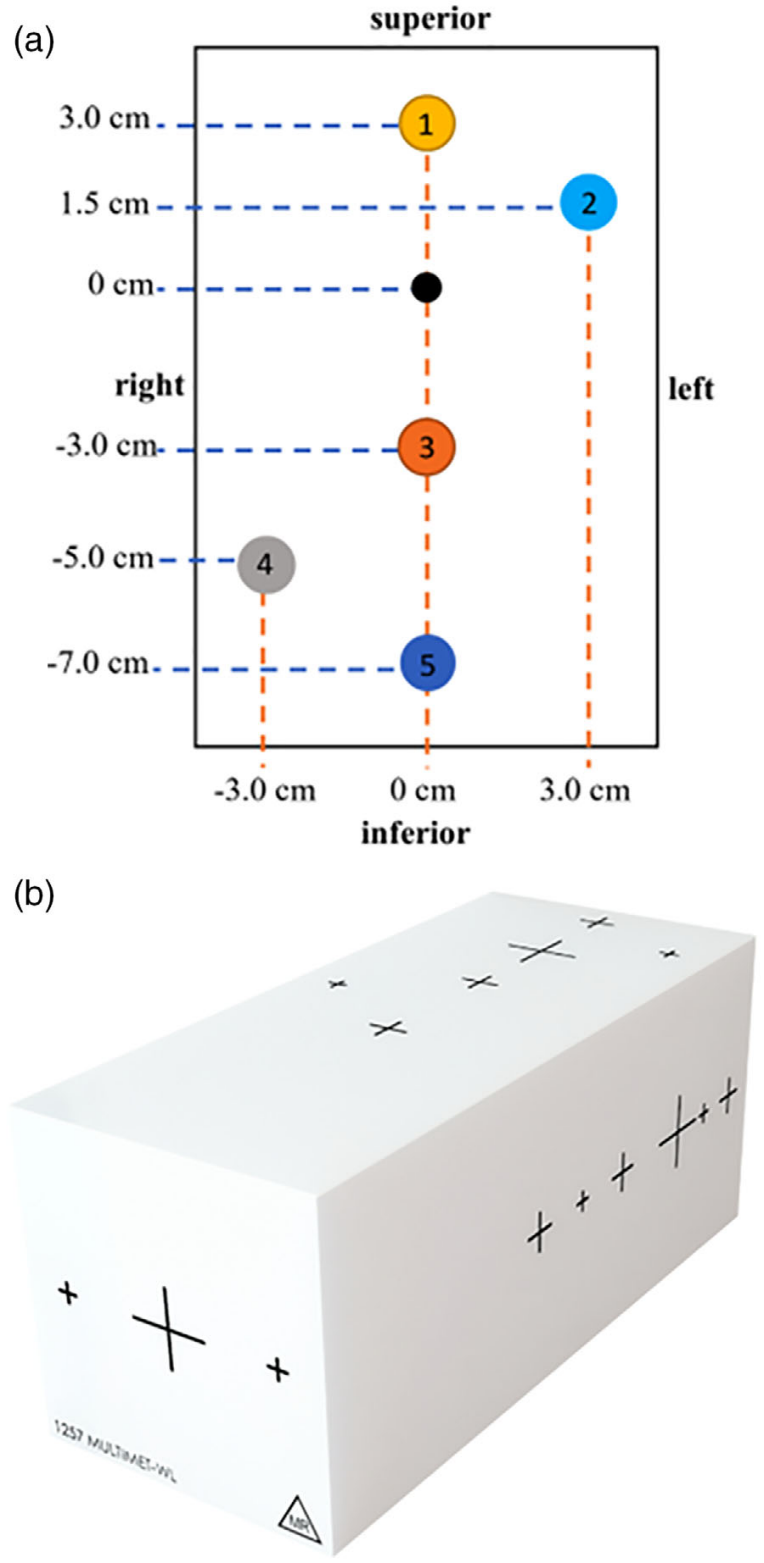
(a) Beams eye view ball bearing positions within the MuliMet‐WL cube, (b) external view of the multiMet‐WL cube. Crosshairs on the external surface indicate ball bearing positions within the cube.

Testing was conducted using the Sun Nuclear (Melbourne, Florida, USA) StereoPHAN phantom and MulitMet‐WL cube (Figure 1).^6^ The MultiMet‐WL phantom is an 8.5 × 8.5 × 12.75 cm^3^ acrylic rectangular prism and contains a set of central‐axis and off‐axis targets set at precise locations. The ball bearing targets are made from tungsten carbide and measure 5.0 ± 0.025 mm. The numbering of the targets shown in Figure 1 is used subsequently when referring to the targets throughout the remainder of the manuscript.

**FIGURE 2:**
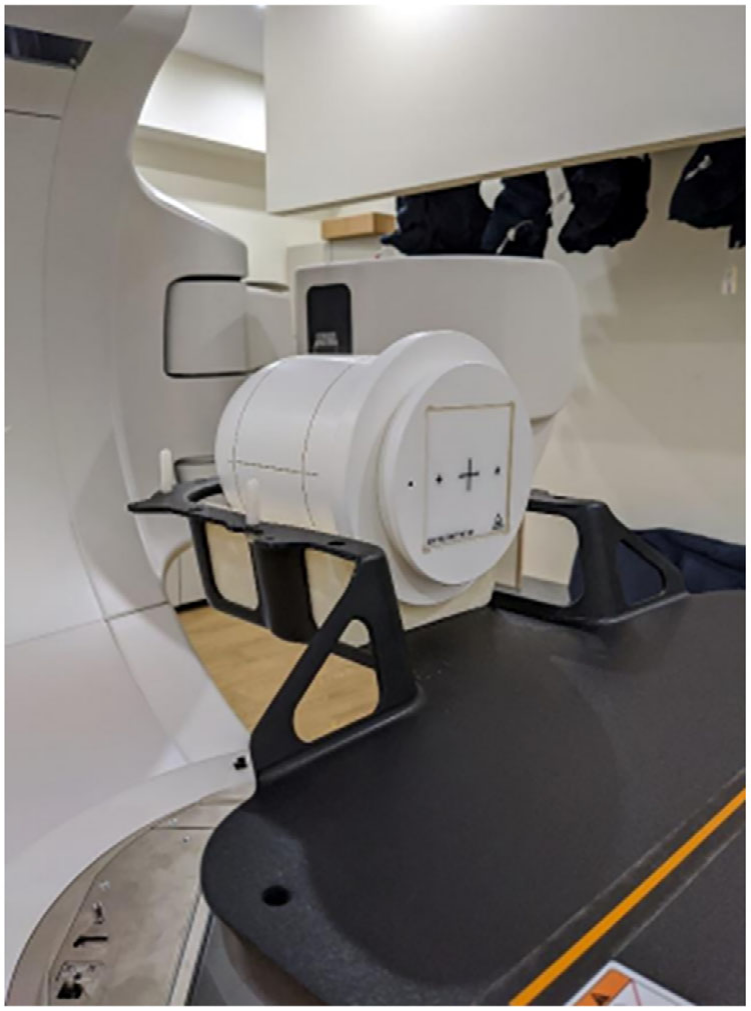
Setup of multiMet‐WL cube within the StereoPhan phantom for off‐axis Winston‐Lutz test measurements.

The WL‐cube is designed to be compatible with the StereoPHAN allowing off‐axis WL testing as well as determining rotational gantry, collimator and couch errors (Figure 2). MultiMet‐WL can be used as a standalone phantom to assure accuracy for off‐axis SRS targets or paired with StereoPHAN for an end‐to‐end stereotactic QA program.

### 2.2 Off‐axis Winston‐Lutz test workflow

The WL‐cube was placed inside the StereoPHAN and positioned on the treatment couch using an in‐house designed and fabricated custom foam cradle designed to fit the phantom within the QFix Encompass SRS headboard (Type RT‐4600‐01, QFix, Avondale, PA) (Figure 2). Initial alignment to the machine isocenter was performed using room lasers and surface markers. A cone‐beam CT (CBCT) scan with 1‐mm slice thickness was acquired, and a six degrees of freedom couch was used to manually match the acquired CBCT image with a previously obtained reference CT, also with a 1‐mm slice thickness.

The electronic portal imaging device (EPID) was used to capture images of the ball‐bearing positions (image resolution 0.39 mm), following a treatment plan in Eclipse (v15.6, Varian Medical Systems, Palo Alto, CA) provided with the MultiMet‐WL phantom. The plan delivered 2 × 2 cm^2^ MLC‐defined beams with a 6 MV flattening filter free (FFF) beam to each target at eight combinations of gantry, couch, and collimator angles. For coplanar target positioning at gantry angles of 90° and 270°, two images of a subset of targets were acquired to prevent overlap.

The results of the off‐axis Winston‐Lutz test were evaluated using the MultiMet‐WL Analysis tool (v2.1.0, Sun Nuclear Corporation, Melbourne, Florida, USA). The analyzer processed the set of DICOM images obtained from each test and calculated the positioning error for each individual target. The analysis assessed the discrepancy between the actual and expected target positions in three ways: (1) the 1D *x*‐ and *y*‐directions for each image, (2) the 2D positional error in each image (vector distance between the center of the MLC defined field and center of ball bearing target), and (3) the 3D positional vector error based on data from all eight images. For the test to be considered acceptable, discrepancies in all measurements had to be less than 1.00 mm.^16^ The MultiMet‐WL Analysis software employs sub‐pixel centroid algorithms, enabling measurement resolution < 0.39 mm, however the exact resolution is not specified by the vendor in the product documentation. The OAWLT workflow was repeated weekly in our department on our three varian TrueBeam linear accelerators fitted with Millenium 120 multi‐leaf collimators from the period of July–December 2024.

### 2.3 Varian TrueBeam machine performance check

During the same measurement period, the varian machine performance check (MPC) was performed on a daily basis using the enhanced couch option. Varian's MPC is an integrated, automated tool designed to assess the critical functions of TrueBeam linear accelerators. Utilizing image‐based evaluations, MPC conducts both beam constancy and geometric performance tests. The beam constancy checks involve acquiring a single megavoltage (MV) image per beam energy to assess parameters such as output, uniformity, and beam center, comparing them against user‐defined baselines. Geometric tests employ a series of kilovoltage (kV) and 6 MV images of a phantom, previously aligned to the room lasers to evaluate aspects like treatment isocenter size, the coincidence of MV and kV isocenters, and the accuracy of collimator and gantry angles.

To compare with the results obtained from the routine OAWLT, four parameters from enhanced couch MPC were selected and analyzed based on their relevance to the OAWLT.^11^

#### 2.3.1 Isocenter size

This parameter measures the spatial accuracy of the treatment isocenter, which is the point in space where the radiation beams converge. It assesses the mechanical and radiation isocenter of the machine by analyzing deviations from the expected position during gantry, collimator, and couch rotations. A larger than expected isocenter size can indicate mechanical misalignments, affecting treatment accuracy.

#### 2.3.2 kV Isocenter offset

This parameter evaluates the deviation between the expected and actual positions of the kilovoltage (kV) imaging system's isocenter. It ensures that the kV imaging system aligns correctly with the megavoltage (MV) treatment isocenter. A misalignment may affect image‐guided radiotherapy (IGRT) accuracy, potentially leading to targeting errors.

#### 2.3.3 Beam center

This parameter measures the center of the radiation beam relative to a reference baseline, verifying that the beam is being delivered at the intended coordinates. It helps detect issues such as beam steering deviations or changes in beam symmetry and energy output. A shift in beam center could indicate a problem with the accelerator's steering coils or beam alignment.

#### 2.3.4 Radiation‐induced couch shift

This parameter evaluates the positional deviation of the treatment couch due to radiation exposure. It helps detect any movement of the couch caused by mechanical instability or radiation‐induced changes. Ensuring couch stability is crucial for maintaining patient positioning accuracy throughout treatment.

## 3 RESULTS

**FIGURE 3:**
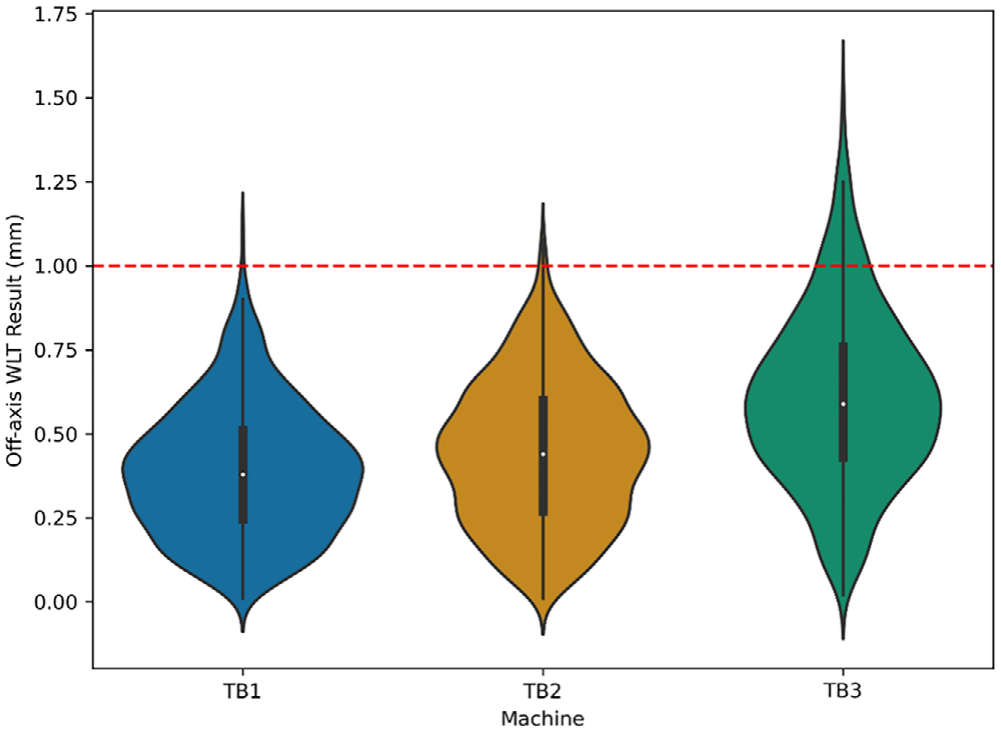
All off‐axis Winston‐Lutz test results for TrueBeam 1 (TB1), TrueBeam 2 (TB2), and TrueBeam 3 (TB3) linear accelerators used in this study. The dashed red line represents the tolerance of 1 mm. The colored areas show the distribution (density) of off‐axis WLT results for each machine, with wider sections indicating more frequent values.

The OAWLT results are summarized in Figure 3 organized by linear accelerator. The median (range) OAWLT result was 0.38 mm (0.01–1.12 mm), 0.44 mm (0.01–1.08 mm), and 0.59 mm (0.02–1.54 mm) for TrueBeam 1 (TB1), TrueBeam 2 (TB2), and TrueBeam 3 (TB3), respectively. The percentage of measurements failing to meet the 1 mm criteria for the OAWLT was 0.2%, 0.4%, and 7.5% for TB1, TB2, and TB3, respectively. The median (range) time to perform the test on the linac was 17 (14–21) min.

Table 1 shows the results further discretized by target location. Across all three linear accelerators, the target with the worst OAWLT result was target 5 (7 cm off‐axis).

**TABLE 1:** The field target coincidence in mm for each specific ball bearing in MultiMet‐WL cube. The data in each cell is organized using the following values: Mean(minimum‐maximum). TB1 = TrueBeam 1, TB2 = TrueBeam 2, TB3 = TrueBeam 3.

	Target location					
Machine	Iso	1	2	3	4	5
TB1	0.34 (0.01–0.78)	0.40 (0.09–0.93)	0.33 (0.05–0.81)	0.40 (0.03–0.94)	0.34 (0.03–0.82)	0.50 (0.02–1.12)
TB2	0.41 (0.04–0.96)	0.42 (0.01–1.05)	0.40 (0.04–0.98)	0.47 (0.05–0.85)	0.37 (0.05–0.8)	0.58 (0.15–1.08)
TB3	0.56 (0.05–1.22)	0.63 (0.05–1.17)	0.49 (0.01–1.07)	0.57 (0.02–1.29)	0.64 (0.09–1.32)	0.68 (0.02–1.54)

**FIGURE 4:**
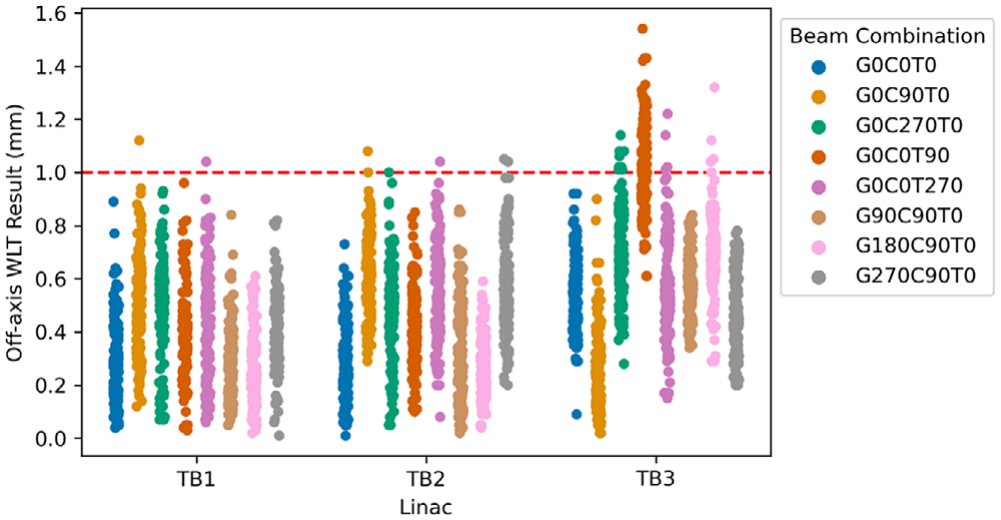
Off‐axis Winston‐Lutz test results for TrueBeam 1 (TB1), TrueBeam 2 (TB2), and TrueBeam 3 (TB3) linear accelerators discretized by beam orientation. The dashed red line represents the tolerance of 1 mm. G = gantry, C = collimator, T = table.

Figure 4 and Table 2 show the results this time discretized by the beam orientation (defined by gantry, collimator, and couch angle) combined across all ball bearing targets.

**TABLE 2:** The field target coincidence in mm for each beam orientation. The data in each cell is organized using the following values: Mean(minimum‐maximum). G = gantry, C = collimator, T = table. TB1 = TrueBeam 1, TB2 = TrueBeam 2, TB3 = TrueBeam 3.

	Beam orientation							
Machine	G0C0 T0	G0C90 T0	G0C270 T0	G0C0 T90	G0C0 T270	G90C90 T0	G180C90 T0	G270C90 T0
TB1	0.30 (0.04–0.89)	0.50 (0.12–1.12)	0.49 (0.07–0.93)	0.41 (0.03–0.96)	0.44 (0.06–1.04)	0.30 (0.05–0.84)	0.27 (0.02–0.61)	0.39 (0.01–0.82)
TB2	0.27 (0.17–0.43)	0.63 (0.40–0.80)	0.47 (0.33–0.66)	0.41 (0.30–0.53)	0.56 (0.37–0.74)	0.35 (0.27–0.47)	0.26 (0.10–0.44)	0.58 (0.43–0.79)
TB3	0.55 (0.45–0.74)	0.27 (0.14–0.46)	0.71 (0.52–0.92)	1.01 (0.76–1.19)	0.56 (0.36–0.69)	0.57 (0.44–0.68)	0.70 (0.51–0.81)	0.47 (0.33–0.59)

**FIGURE 5 (:**
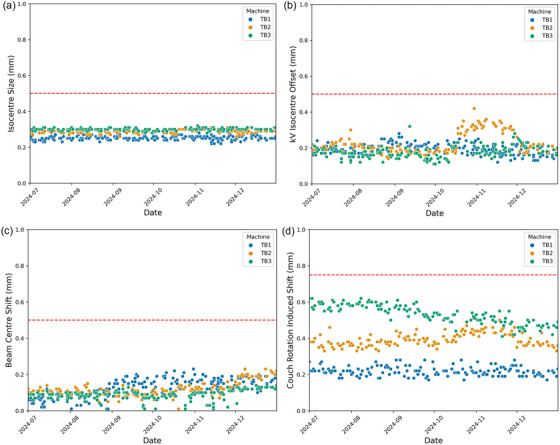
a) Varian machine performance check isocenter size parameter during the study period. Dashed red line indicates Varian tolerance. TB1 = TrueBeam 1, TB2 = TrueBeam2, TB3 = TrueBeam3. (b) varian machine performance check kV isocenter offset parameter during the study period. Dashed red line indicates varian tolerance. TB1 = TrueBeam 1, TB2 = TrueBeam2, TB3 = TrueBeam3. (c) Varian machine performance check beam center shift parameter during the study period. Dashed red line indicates Varian tolerance. TB1 = TrueBeam 1, TB2 = TrueBeam2, TB3 = TrueBeam3. (d) Varian machine performance check radiation‐induced couch shift parameter during the study period. Dashed red line indicates Varian tolerance. TB1 = TrueBeam 1, TB2 = TrueBeam2, TB3 = TrueBeam3.

The MPC results for the isocenter size, kV isocenter offset, beam center, and radiation‐induced couch shift during the study period are shown in Figure 5a–d, respectively. The dashed red line on each plot represents the Varian tolerance for each parameter.

Visual inspection of scatter plots comparing OAWLT results to the various MPC parameters in Appendix I did not demonstrate any clear evidence of correlation. The data points appear widely dispersed without an observable linear or monotonic trend. Given the absence of any discernible pattern, formal correlation analyses were not pursued. These findings suggest that, within the scope of this dataset, the MPC parameters may not directly predict OAWLT performance.

## 4 DISCUSSION

Across the 6 month period of weekly OAWLT measurements 0.2%, 0.4%, and 7.5% of the results failed the 1 mm tolerance for TB1, TB2, and TB3 linacs, respectively. Additionally, the maximum OAWLT for TB1, TB2, and TB3 was 1.12, 1.08, and 1.54 mm. For each of the three linacs, the maximum error occurred for target 5, which was 7 cm off axis. For the traditional central‐axis WLT, a 1 mm tolerance is recommended by the American Association for Physicists in Medicine (AAPM).^16^ At the time of publication, no societal recommendations exist for tolerances of the OAWLT, however the recently published AAPM‐RSS Medical Physics Practice Guideline 9.b recommends that each institute develop their own OAWLT assessment policy based on available publications.^17^ It is hoped that this study will contribute to that body of work. The AAPM Task Group 362: Multi‐lesion Stereotactic Radiosurgery is currently drafting guidelines which may give recommendations on OAWLT frequency and tolerances.

Across the three linacs studied in this publication, one linac (TB3) was an outlier. This linac is the oldest of the three in the department (7 years) and the sub‐optimal results were largely related to beam orientations for non‐zero couch angles, with the couch 90° beam in particular giving the worst results. As a result of this study, SRS treatments were no longer recommended on this linac in our department. This was made possible via the three beam matched machines in our department. If this was not possible in other departments, alternative measures such as using couch 270° rather than couch 90° for the vertex fields in SRS plans could be made. Alternatively, GTV‐PTV margins may be adjusted to account for the additional uncertainties.

The WL‐cube was also utilized by Gao et al.^7^ across seven linacs, and found that all results were achievable to within a 1 mm tolerance for up to 7 cm off‐axis. However, one important distinction in workflow between this current study and that by Gao et al. must be made. In this present study, the WL‐cube was aligned to the kV isocenter using CBCT, mimicking the patient setup workflow for SRS treatments. Whereas in the study by Gao et al. EPID images were acquired to align the phantom to the MV isocenter. Therefore, an additional uncertainty in kV‐MV isocenter alignment is captured in this current study, that is not captured in the one by Gao et al. The authors of this current study believe that it is essential to capture this uncertainty in the OAWLT procedure such that it is more representative of patient alignment for SIMT SRS treatments. Nonetheless, the study by Gao et al. also showed that there is a clear trend with worse off‐axis target positioning as the distance from linac isocenter is increased, and that there is a machine dependence of the results, with newer machines performing better than their older counterparts.^7^ The trend for worse off‐axis target positioning and machine dependence was also found in the recent study by Capaldi et al.^18^

From the data shown in Appendix I, there does not appear to be any clear correlation between the OAWLT and parameters reported by MPC. Firstly, the MPC procedure utilizes a 6 MV beam to perform the geometry tests, whereas a 6 MV FFF beam was utilized for the OAWLT procedure in this study. A 6 MV FFF beam energy was chosen to replicate the beam energy utilized for SIMT SRS patient treatments in our department. The use of a different beam energy might hinder correlation between the results due to the differences in beam positioning between the two energies. Secondly, the parameters reported by MPC report only on limited characteristics of the target accuracy of the machine. For example, the kV‐Isocenter Offset parameter does not include beam acquisitions for non‐zero couch angles and is an average value across gantry and collimator angles. The radiation‐induced couch shift parameter measures only the maximum deviation between averaged couch zero and non‐couch zero beam acquisitions during MPC. Finally, the attempted correlation analysis between the two datasets may be limited by the small sample size used during this study.

Whilst this study exhibits the ability of the routine OAWLT to detect deviations that could indicate target positioning issues in a clinical setting, it does not prove its sensitivity to detecting known errors in target positioning. However, this was previously investigated by Pudsey et al.^6^ who showed that the OAWLT was sensitive to detecting intentionally introduced errors in collimator, gantry, and couch rotation. The study also found that the sensitivity of the OAWLT in detecting these rotational errors was superior to patient specific quality assurance (PSQA) fluence measurements with the Sun Nuclear SRS MapCheck 2D diode array, and that these rotational errors caused SIMT SRS plans to become clinically unacceptable before violating routine machine QA limits recommended by the AAPM Task Group 142 report.^16^ Based on the variability observed in our results, along with those by Gao et al.^7^ and Capaldi et al.^18^ we suggest that departments offering a SIMT SRS program consider performing an OAWLT on a weekly basis. While our data do not prescribe an exact frequency, a weekly interval offers a pragmatic balance between early detection of off‐axis deviations and clinical resource demands. By combining a weekly OAWLT with strict regular machine quality assurance, routine PSQA fluence measurements, and robust treatment planning methods,^19^ departments may deliver SIMT SRS with confidence in their off‐axis target positioning.

This study demonstrates the utility of a routine OAWLT or Varian TrueBeam linacs. However, one limitation of the study is that the results may not be generalized to other linac types. Future studies will focus on collecting long‐term OAWLT data on several linac types to examine the generalizability of the results.

## 5 CONCLUSIONS

Routine OAWLT measurements on three Varian TrueBeam linacs were performed. The maximum OAWLT error was found to be 1.12, 1.08, and 1.54 mm, for each of the three linacs. Off‐axis target positioning was observed to increase with distance from linac isocenter. A 1 mm tolerance for routine OAWLT may be achievable on Varian TrueBeam linacs with strict routine machine QA (e.g. regular IsoCal procedures). Given that the median time to perform the test on the linac was 17 min, it is recommended that an OAWLT be performed on a weekly basis by all departments offering a SIMT SRS program. Supporting Information

## AUTHOR CONTRIBUTIONS

J.P. was responsible for conception and organization of the study, manuscript writing and editing, R.B. was responsible for collecting data and manuscript writing and editing, R.C. was responsible for collecting data and manuscript writing and editing, H.P. was responsible for data collection, D.K. was responsible for collecting data and manuscript writing and editing, J.Y. was responsible for manuscript writing and editing, R.Q. was responsible for collecting data and manuscript writing and editing, RG was responsible for collecting data and manuscript writing and editing, A.H. was responsible for collecting data and manuscript writing and editing.

## CONFLICT OF INTEREST STATEMENT

The authors declare no conflicts of interest.

## Supporting information



Supporting Information
