# Supplementary material for: Quality assurance of single isocentre multiple target stereotactic radiosurgery: Findings from long‐term off‐axis Winston‐Lutz testing and machine performance checks
Source: J Appl Clin Med Phys. 2025 Sep 30;26(10):e70275. doi: 10.1002/acm2.70275 (PMC12483765; doi:10.1002/acm2.70275)
Supplement: Supplementary file 1 — Supporting Information [file ACM2-26-e70275-s001.docx]

**
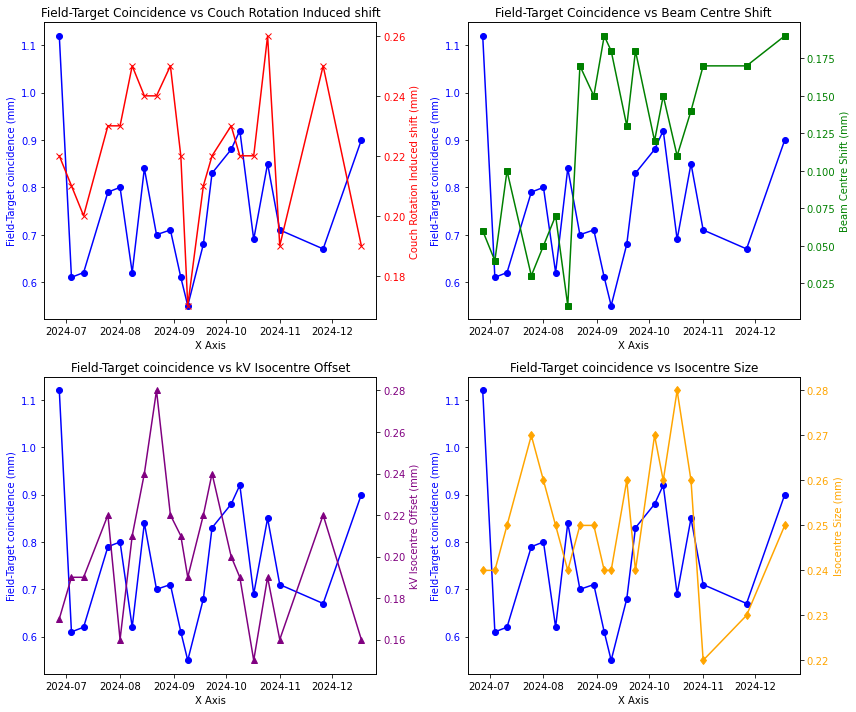
**

**TB1 comparison of OAWLT field-target coincidence vs. MPC parameters throughout the duration of the study. The reported field-target coincidence is the maximum error found for that measurement session.**

**
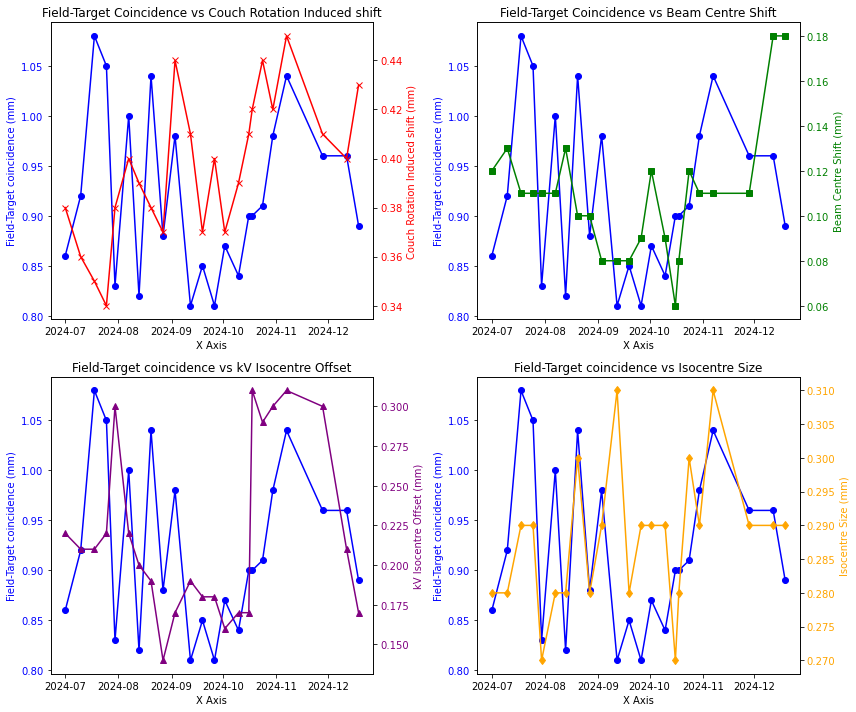
**

**TB2 comparison of OAWLT field-target coincidence vs. MPC parameters throughout the duration of the study. The reported field-target coincidence is the maximum error found for that measurement session.**

**
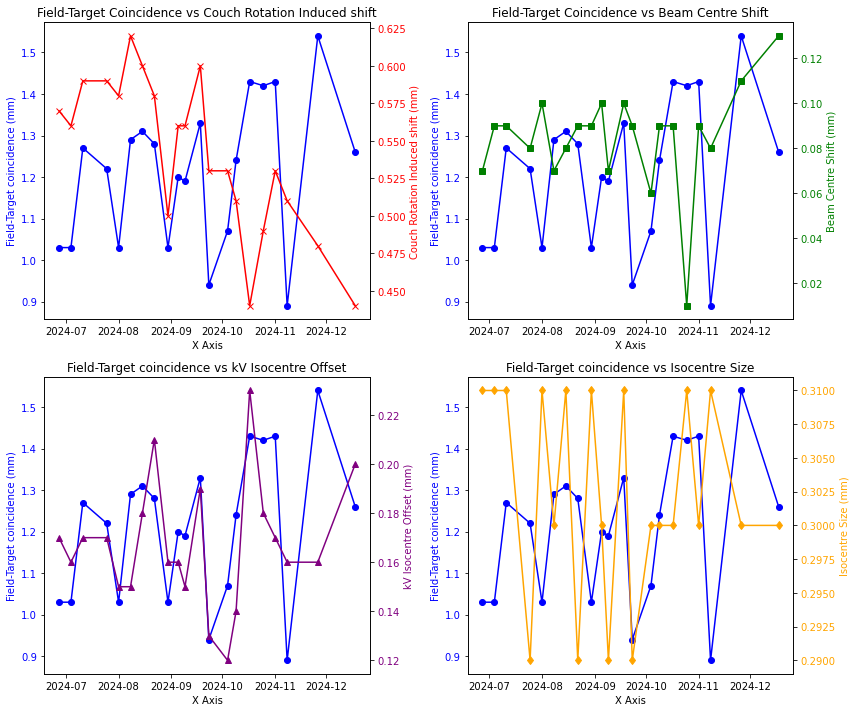
**

**TB3 comparison of OAWLT field-target coincidence vs. MPC parameters throughout the duration of the study. The reported field-target coincidence is the maximum error found for that measurement session.**
